# Neurovascular coupling during dynamic upper body resistance exercise in healthy individuals

**DOI:** 10.1113/EP091970

**Published:** 2024-09-25

**Authors:** Stephanie Korad, Toby Mündel, Blake G. Perry

**Affiliations:** ^1^ School of Health Sciences Massey University Wellington New Zealand; ^2^ School of Sport, Exercise and Nutrition Massey University Palmerston North New Zealand; ^3^ Department of Kinesiology Brock University St Catharines Ontario Canada

**Keywords:** middle cerebral artery blood velocity, neurovascular coupling, resistance exercise

## Abstract

During unilateral static and rhythmic handgrip exercise, middle cerebral artery blood velocity (MCAv) increases in the contralateral side to the exercising limb. However, whether this neurovascular coupling‐mediated increase in contralateral MCAv is apparent against a background of fluctuating perfusion pressure produced by dynamic resistance exercise (RE) is unclear. We examined the cerebral haemodynamic response to unilateral dynamic RE in 30 healthy individuals (female = 16, mean ± SD: age, 26 ± 6 years; height, 175 ± 10 cm; weight, 74 ± 15 kg; body mass index, 24 ± 5 kg m^−2^). Participants completed four sets of 10 paced repetitions (15 repetitions min^−1^) of unilateral bicep curl exercise at 60% of the predicted one‐repetition maximum (7 ± 3 kg). Beat‐to‐beat blood pressure, bilateral MCAv and end‐tidal carbon dioxide were measured throughout. One‐way ANOVA was used to analyse cardiovascular variables and two‐way ANOVA to analyse dependent cerebrovascular variables (side × sets, 2 × 5). A linear mixed model analysis was also performed to investigate the effects of end‐tidal carbon dioxide and mean arterial blood pressure on MCAv. In comparison to baseline, within‐exercise mean arterial blood pressure increased (*P *< 0.001) across the sets, whereas bilateral MCAv decreased (*P *< 0.001). However, no significant interaction effect was observed for any dependent variables (all *P *> 0.787). The linear mixed model revealed that end‐tidal carbon dioxide had the greatest effect on MCAv (estimate = 1.019, *t* = 8.490, *P* < 0.001). No differences were seen in contralateral and ipsilateral MCAv during dynamic RE, suggesting that neurovascular coupling contributions during dynamic RE might be masked by other regulators, such as blood pressure.

## Abstract (highlights)

- **What is the central question of this study?**
  During unilateral static resistance exercise (RE), neurovascular coupling mediates an increase in cerebral blood flow in the contralateral hemisphere. Whether this response persists during dynamic RE, with concomitant large fluctuations in blood pressure, remains unclear.
- **What is the main finding and its importance?**
  During unilateral dynamic RE, there was no difference in blood velocity between the contralateral and ipsilateral middle cerebral arteries. The presence of bilateral fluctuations in blood velocity during unilateral dynamic RE could increase bilateral shear stress and confer beneficial vascular adaptations in both cerebral hemispheres.

## 1 INTRODUCTION

Cerebral haemodynamics during dynamic exercise are complicated because the many regulators of cerebral blood flow (CBF) are perturbed concomitantly. These regulators include neurovascular coupling (NVC), arterial carbon dioxide content, arterial blood pressure, sympathetic nerve activity and cerebral autoregulation (CA) (Willie et al., 2014). Neurovascular coupling refers to the matching of perfusion to tissue activity, whereby increased neuronal activity mediates a rise in local CBF. During unilateral static handgrip exercise at low intensities (30%–40% of maximal voluntary contraction), NVC mediates an increase in blood velocity in the middle cerebral artery (MCAv, as a proxy for CBF) contralateral to the exercising limb by 14%–18% (Braz et al., 2014; Imms et al., 1998) and an increase in contralateral internal carotid artery (ICA) blood flow by 14%–22% (Fernandes et al., 2016; Hirasawa et al., 2016). Likewise, during unilateral rhythmic handgrip exercise (30 contractions min^−1^ for 5 min), Jorgensen et al. (1993) reported an increase in MCAv contralateral to the exercising limb only, whereas others have reported a bilateral increase in MCAv, with slightly greater increases in contralateral MCAv (Giller et al., 2000; Ide et al., 1998). Collectively, these data indicate that low‐intensity static resistance exercise (RE) increases CBF, primarily in the hemisphere contralateral to the exercising limb.

Dynamic RE is characterized by cyclic concentric and eccentric skeletal muscle contractions, generating sinusoidal fluctuations in blood pressure that are transmitted to the cerebral circulation (Perry & Lucas, 2021). Consequently, the MCAv profile during dynamic RE reflects arterial blood pressure. Although rare, the dynamic RE‐induced rapid changes in blood pressure and cerebral perfusion pressure can cause cerebrovascular injury (Edwards et al., 2002). Given the wide range of benefits that RE confers (e.g., increased muscle mass and strength), dynamic RE is recommended during the recovery from stroke and can improve quality of life (Ali et al., 2021). Consideration is required when prescribing RE following a stroke because CA is impaired globally (Aries et al., 2010). Thus, the correct prescription of RE in this population is crucial in limiting exposure to extreme blood pressures to avoid further cerebrovascular injury, yet still be sufficiently stimulating to permit the advantageous adaptations that RE confers.

The plethora of CBF regulators still exert individual control during RE despite the background of rapidly fluctuating perfusion pressure (Perry & Lucas, 2021). However, the contribution of NVC to the MCAv profile within dynamic RE is yet to be determined. The results of the present study could inform the safe prescription of dynamic RE, particularly for individuals who have experienced cerebrovascular injury. In the present study, we investigate the cerebral haemodynamic response to unilateral dynamic upper body RE in healthy individuals. We hypothesize that during unilateral dynamic RE, there will be no differences between contralateral and ipsilateral MCAv because mean arterial pressure (MAP) and the partial pressure of end‐tidal carbon dioxide ($P_{\mathrm{ET},CO_{2}}$) will override NVC.

## 2 MATERIALS AND METHODS

### 2.1 Ethics and informed consent

Participants were informed of the experimental procedures and aware of the purpose of this research, in addition to the potential risk associated with participating. Written informed consent was provided by the participants prior to partaking in the research. The study was approved by the Massey University Human Ethics Committee (SOA 21/22) and in agreement with the latest version of the *Declaration of Helsinki* apart from registration in a database.

### 2.2 Participants

An a priori power analysis (G*Power v.3.1.9.4; Heinrich Heine University Düsseldorf, Düsseldorf, Germany) was conducted using data from the study by Jorgensen et al. (1993) with similar outcome measures (i.e., MCAv). Based on conventional α (0.05) and β (0.80) values, a minimum of 23 participants were required. A total of 30 participants (female = 16) were recruited for this study (mean ± SD: age, 26 ± 6 years; height, 175 ± 10 cm; weight, 74 ± 15 kg; body mass index, 24 ± 5 kg m^−2^). All participants were healthy and free of any medical conditions, were not taking any form of medication other than oral contraception (*n* = 4) or an intrauterine device (*n* = 1), were non‐smokers and had no history or symptoms of cardiovascular, pulmonary, metabolic or neurological disease. Female participants self‐reported their menstrual cycle phase, with visits occurring during the early follicular phase (low oestrogen and progesterone) and during the placebo phase for those using oral contraceptives. Our previous findings have indicated no functional differences in cerebrovascular responses to acute changes in MAP between menstrual cycle phases (Korad et al., 2022). A mixed cohort was used for this study, with some participants having some experience with RE.

### 2.3 Study design

Participants visited the temperature‐controlled laboratory twice, first for familiarization and second for the experimental trial. Explanation and demonstration of the equipment, procedures and risk of participation were communicated during the familiarization. Once consent was provided, bilateral middle cerebral arteries were insonated for the measurement of MCAv as described below. The participant's unilateral bicep curl one‐repetition maximum (1RM, dominant arm) was estimated during familiarization using the equation of Brzycki (1993). Pilot testing revealed that a bicep curl: (1) requires little familiarization for correct technique; (2) generates sinusoidal fluctuations in blood pressure despite the small muscle mass; (3) permits the concurrent measurement of blood pressure and isolation of the non‐active limbs; and (4) recruits similar musculature to the existing literature on this topic. The working intensity for the trial was 60% of the predicted 1RM (60%1RM, mean ± SD: bicep curl predicted 1RM 12 ± 5 kg; 60%1RM, 7 ± 3 kg). The participants practised the bicep curl at 60%1RM whilst maintaining the contraction tempo and breathing pattern outlined below.

### 2.4 Experimental protocol

**FIGURE 1:**
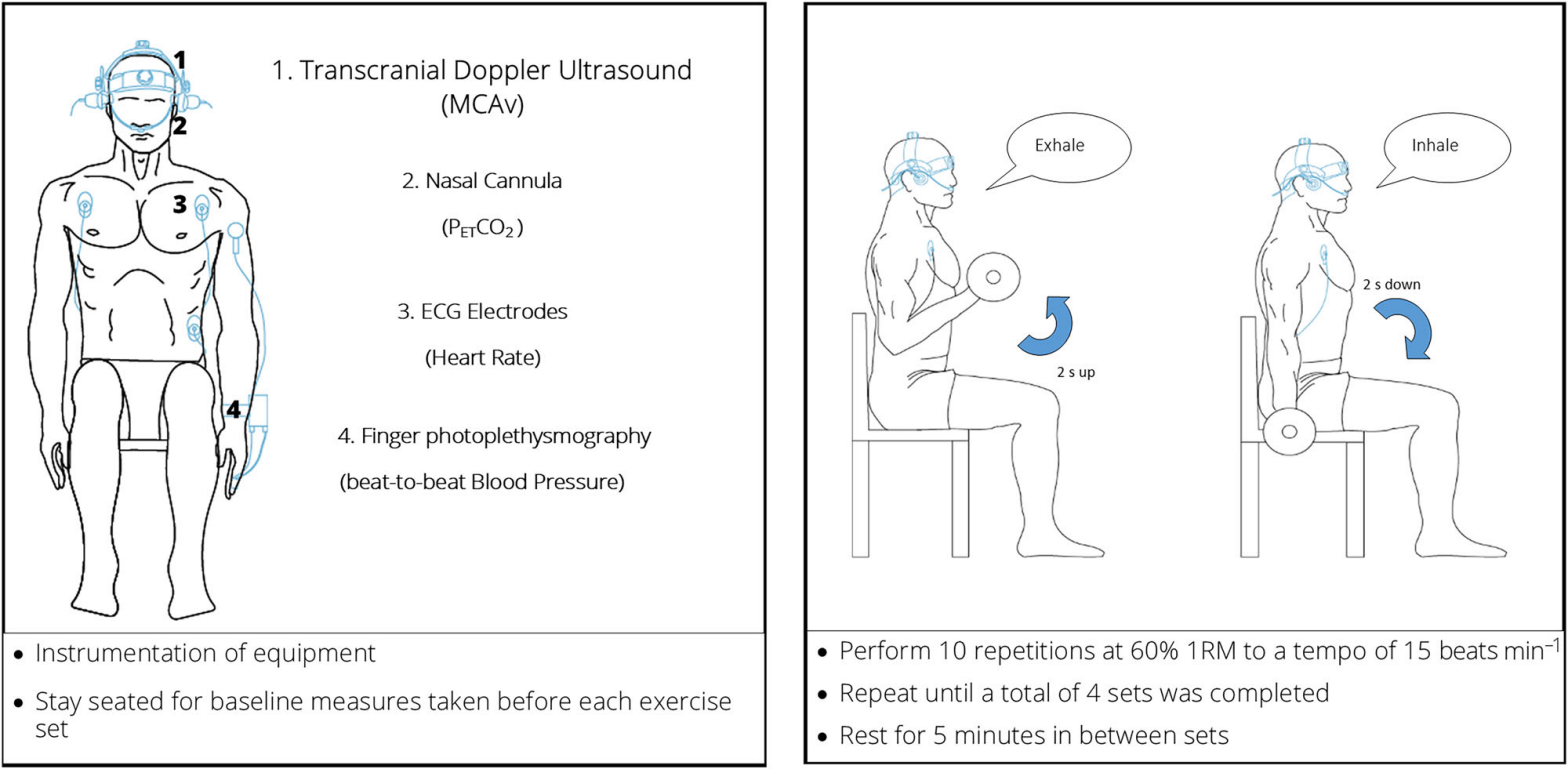
Experimental protocol. The exercise sets consisted of 10 repetitions of bicep curls. Haemodynamic variables (middle cerebral artery blood velocity, blood pressure and heart rate) and partial pressure of end‐tidal carbon dioxide ($P_{\mathrm{ET},CO_{2}}$) were measured throughout.

The familiarization occurred >1 week before the trial. Participants arrived at the laboratory having refrained from caffeinated beverages for 12 h and from vigorous exercise and alcohol consumption for ≥24 h prior to testing. The participant was instructed to consume 500 mL of water the night before and 500 mL ∼4 h before the experiment. An overview of the experiment is shown in Figure 1.

A urine sample for analysis of urine specific gravity was provided by the participant on arrival to ensure euhydration (urine specific gravity < 1.020), then the participant was seated for instrumentation. Once instrumented, the participant rested for 20 min. Pre‐exercise baseline values were recorded for 5 min and immediately preceding each exercise set thereafter. During exercise, the participant performed 10 repetitions of unilateral bicep curl at 60%1RM to a tempo of 15 beats min^−1^ (a repetition cycle length of 4 s; 2 s per concentric and eccentric phase) as timed by a metronome. Exercise data were averaged across all 10 repetitions. Participants exhaled during the concentric phase and inhaled during the eccentric phase, avoiding the Valsalva manoeuvre. The participant then rested for 5 min, and baseline measures were taken in the last minute of this rest period. The sequence was repeated until a total of four sets were completed. The participant was reminded of the breathing technique, and to avoid the Valsalva manoeuvre, before each set.

### 2.5 Systemic haemodynamics

Heart rate (HR) was measured using a three‐lead ECG (ADInstruments, Australia). Non‐invasive beat‐to‐beat arterial blood pressure was measured by finger photoplethysmography (Finapres Medical Systems, The Netherlands). The cuff was placed on the middle phalanx of either the middle finger or the index finger on the non‐dominant hand and referenced to the level of the heart using the height‐correction unit. Blood pressure values were checked against an automated sphygmomanometer (Suresigns VM4, Philips Medical Systems, Philips, The Netherlands) during baseline and 2 min following each exercise bout and corrected when necessary.

### 2.6 Middle cerebral artery blood velocity

Bilateral MCAv was measured using transcranial Doppler (TCD) ultrasonography (Doppler‐Box X, DWL, Compumedics, Germany). Using an adjustable headband, a 2 MHz probe was fixed in position over the temporal window, above the zygomatic arch. The M1 segment of the middle cerebral artery was identified using search techniques described elsewhere (Willie et al., 2011). Ultrasound gel (Tensive, Parker Laboratory, Fairfield, NY, USA) was placed between the transducer probe and the skin to ascertain the highest quality signal.

### 2.7 Partial pressure of end‐tidal carbon dioxide

The $P_{\mathrm{ET},CO_{2}}$ was measured using a calibrated online gas analyser (ML206 Gas Analyser, ADInstruments, Australia) and was collected throughout using a nasal cannula.

### 2.8 Urine analysis

Given that hydration status can influence cerebrovascular regulation (Perry et al., 2016), urine specific gravity was measured using a hand‐held refractometer (Atgo Co., Tokyo, Japan) ∼30 min prior to the trial to verify hydration status (mean ± SD, 1.010 ± 0.007), with all participants hydrated on first measurement of urine specific gravity.

### 2.9 Data acquisition

All data were collected continuously using an analog‐to‐digital converter (PowerLab, ADInstruments, Australia) interfaced with a computer, then analysed using LabChart software (v.8.1.13 ADInstruments, Australia).

### 2.10 Data analysis

#### 2.10.1 Dependent measures

Mean MCAv (MCAv_mean_) was derived from the mean velocity of the raw MCAv waveform in LabChart. The MAP was calculated using the expression 1/3 systolic blood pressure + 2/3 diastolic blood pressure. The cerebrovascular conductance index (CVCi) was calculated using the equation CVCi = MCAv_mean_/MAP. The SMCAv (the maximum blood velocity in the MCA during systole) and the DMCAv (the minimum blood velocity in the MCA during diastole) were used to calculate the Gosling pulsatility index (PI) for the MCA (SMCAv − DMCAv/MCAv_mean_) (Gosling & King, 1974).

#### 2.10.2 Statistical analysis

Data were analysed using SPSS statistical software v.28 (IBM Corp., Armonk, NY, USA). Statistical significance was set at *P ≤* 0.05. The Shapiro–Wilk test confirmed that all data were normally distributed (*P > *0.081). To check for drift in physiological variables of interest before exercise, a two‐way ANOVA was performed to analyse cerebrovascular variables in the baseline period immediately prior to each set of exercise (side × baselines, 2 × 5), and a one‐way ANOVA was performed to analyse cardiovascular baseline data. Cerebrovascular dependent variables of interest at initial baseline (following instrumentation at the beginning of the trial) and during dynamic RE (average data across all performed repetitions) were analysed using a two‐way ANOVA [side × sets (including initial baseline), 2 × 5], whereas cardiovascular data were analysed using a one‐way ANOVA. Partial η^2^ was reported for the interaction effect only. Cohen (2013) recognized large effect sizes as >0.1379, medium as 0.0588–0.1379 and small as <0.0099. All data are displayed as the mean ± SD. A linear mixed model analysis was also performed to investigate the effects of $P_{\mathrm{ET},CO_{2}}$ and MAP on MCAv. The analysis included a random effect for participant to control for within‐subject correlations. Fixed effects included $P_{\mathrm{ET},CO_{2}}$ MAP, sets and MCAv side. Four models were analysed: a baseline model; $P_{\mathrm{ET},CO_{2}}$ only; MAP only; and a combined $P_{\mathrm{ET},CO_{2}}$ and MAP model. Type III test of fixed effect and estimates of fixed effects were examined.

## 3 RESULTS

### 3.1 Baseline measurements

Baseline measures across the trial are shown in Table 1 for cardiovascular measures and in Table 2 for cerebrovascular measures. There were no significant differences in baseline data for cerebrovascular measures; however, MAP was increased from initial baseline prior to set 4 (for values, see Table 1).

**TABLE 1:** Mean cardiovascular measures during baseline and within exercise.

Variable	Sets					*P*‐value
	Initial	Set 1	Set 2	Set 3	Set 4	
Baseline (pre‐exercise)						
$P_{\mathrm{ET},CO_{2}}$ (mmHg)	37 ± 5	37 ± 5	36 ± 5	36 ± 5	35 ± 5	0.706
HR (beats min^−1^)	70 ± 13	72 ± 13	70 ± 14	70 ± 13	71 ± 13	0.928
MAP (mmHg)	82 ± 8	87 ± 9	88 ± 9	88 ± 9	91 ± 10^a^	**0.011**
Exercise						
$P_{\mathrm{ET},CO_{2}}$ (mmHg)	37 ± 5	35 ± 5	34 ± 5	33 ± 5^a^, ^b^	33 ± 5^a^, ^b^	**0.005**
HR (beats min^−1^)	70 ± 13	88 ± 15^a^	87 ± 14^a^	88 ± 14^a^	88 ± 15^a^	**<0.001**
MAP (mmHg)	82 ± 8	95 ± 13^a^	96 ± 13^a^	98 ± 14^a^	100 ± 14^a^	**<0.001**

*Note*: Data are presented as the mean ± SD, *n* = 30.

Abbreviations: HR, heart rate; MAP, mean arterial pressure; $P_{\mathrm{ET},CO_{2}}$, end‐tidal carbon dioxide.

^a^ Different from initial baseline (*P* ≤ 0.024).

^b^ Different from set 1 (*P* ≤ 0.002).

**TABLE 2:** Mean cerebrovascular measures during baseline and within exercise.

Variable	Condition		Sets				*P*‐value			Partial ƞ^2^
		Initial	Set 1	Set 2	Set 3	Set 4	Side	Set	Interaction	
Baseline (pre‐exercise)										
MCAv_mean_ (cm s^−1^)	Contralateral	66 ± 11	67 ± 10	66 ± 10	65 ± 10^b^	64 ± 10^a^, ^b^	0.753	**<0.001**	0.677	0.010
	Ipsilateral	66 ± 11	65 ± 10	64 ± 11	64 ± 10^b^	63 ± 11^a^, ^b^				
CVCi (cm s^−1^ mmHg^−1^)	Contralateral	0.81 ± 0.14	0.77 ± 0.14^a^	0.76 ± 0.15^a^, ^b^	0.75 ± 0.14^a^, ^b^	0.71 ± 0.14^a^, ^b^, ^c^, ^d^	0.771	**<0.001**	0.902	0.004
	Ipsilateral	0.80 ± 0.15	0.76 ± 0.14^a^	0.74 ± 0.14^a^, ^b^	0.74 ± 0.14^a^, ^b^	0.71 ± 0.14^a^, ^b^, ^c^, ^d^				
PI	Contralateral	0.82 ± 0.12	0.81 ± 0.13	0.83 ± 0.15	0.82 ± 0.16	0.82 ± 0.16	0.781	0.496	0.997	0.001
	Ipsilateral	0.83 ± 0.13	0.83 ± 0.13	0.84 ± 0.15	0.83 ± 0.15	0.83 ± 0.15				
Exercise										
MCAv_mean_ (cm s^−1^)	Contralateral	66 ± 11	64 ± 9	61 ± 9^a^, ^b^	60 ± 8^a^, ^b^	59 ± 8^a^, ^b^, ^c^	0.633	**<0.001**	0.894	0.005
	Ipsilateral	66 ± 11	62 ± 10	60 ± 9^a^, ^b^	59 ± 9^a^, ^b^	59 ± 10^a^, ^b^, ^c^				
CVCi (cm s^−1^ mmHg^−1^)	Contralateral	0.81 ± 0.14	0.69 ± 0.13^a^	0.65 ± 0.13^a^, ^b^	0.63 ± 0.13^a^, ^b^, ^c^	0.61 ± 0.13^a^, ^b^, ^c^, ^d^	0.716	**<0.001**	0.959	0.003
	Ipsilateral	0.80 ± 0.15	0.67 ± 0.13^a^	0.64 ± 0.13^a^, ^b^	0.62 ± 0.13^a^, ^b^, ^c^	0.60 ± 0.13^a^, ^b^, ^c^, ^d^				
PI	Contralateral	0.82 ± 0.12	0.81 ± 0.14	0.80 ± 0.14	0.80 ± 0.13	0.80 ± 0.13	0.884	0.259	0.996	0.001
	Ipsilateral	0.83 ± 0.13	0.81 ± 0.13	0.80 ± 0.13	0.81 ± 0.13	0.80 ± 0.12				

*Note*: Data are presented as the mean ± SD, *n* = 30.

Abbreviations: CVCi, cerebrovascular conductance index; MCAv_mean_, mean middle cerebral artery blood velocity; PI, pulsatility index.

^a^ Different from initial baseline (*P *≤ 0.020).

^b^ Different from set 1 (*P *≤ 0.007).

^c^ Different from set 2 (*P *≤ 0.050).

^d^ Different from set 3 (*P* ≤ 0.006).

### 3.2 Response to dynamic resistance exercise

**FIGURE 2:**
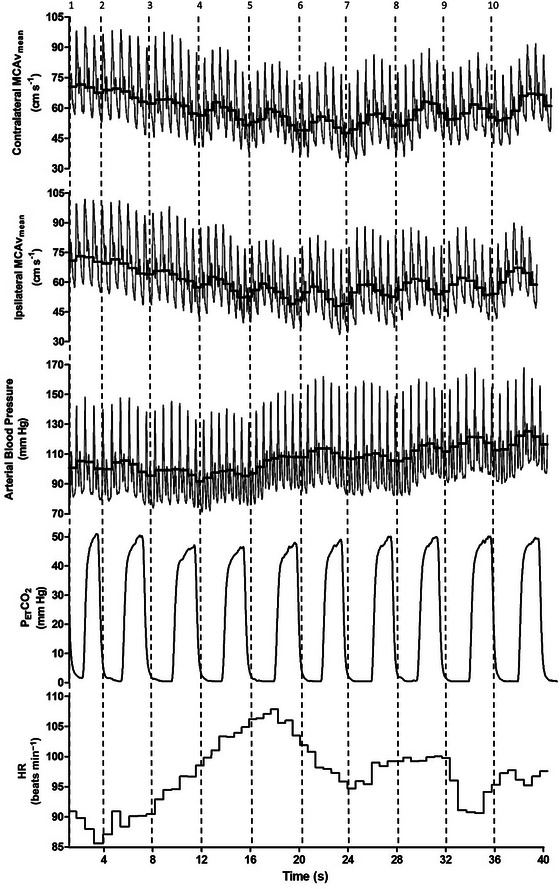
Typical traces of the contralateral and ipsilateral middle cerebral artery blood velocity (MCAv), arterial blood pressure (ABP), partial pressure of end tidal carbon dioxide ($P_{\mathrm{ET},CO_{2}}$) and heart rate (HR) during exercise. The thick black lines in the MCAv and ABP traces represent mean MCAv and mean arterial pressure, respectively. The numbers indicate the repetition count, with the dotted line denoting the start (concentric phase) of each repetition (*n* = 1).

The typical cerebrovascular and cardiovascular responses to exercise are detailed in Figure 2. Mean group within‐exercise cardiovascular responses across the 10 bicep curls are shown in Table 1, with the cerebrovascular responses during the same period shown in Table 2. When examining the cerebrovascular responses to exercise, there were no significant differences between the ipsilateral or contralateral MCAv, CVCi or PI (all *P *> 0.633). However, there was a significant decrease in $P_{\mathrm{ET},CO_{2}}$ in sets 3 and 4 relative to initial baseline and set 1, and the MAP and HR increased compared with initial baseline across all exercise sets (all *P *< 0.005). An effect of set (all *P* < 0.001) indicates that both ipsilateral and contralateral MCAv_mean_ and CVCi values decreased across the sets during exercise (see Table 2).

### 3.3 Linear mixed model analysis

The results of the linear mixed model analysis reflected those of the ANOVA. The baseline model revealed a significant effect of sets (*F* = 14.652, *P* < 0.001) but non‐significant effect of MCAv side (*F* = 2.407, *P* = 0.122). The $P_{\mathrm{ET},CO_{2}}$‐only model showed a strong positive effect of $P_{\mathrm{ET},CO_{2}}$ on MCAv (estimate = 1.027, *t* = 8.537, *P *< 0.001), with a high *F*‐value (72.874), indicating a strong relationship. The MAP‐only model also showed a positive effect of MAP on MCAv (estimate = 0.127, *t* = 2.388, *P *= 0.018). The combined model revealed that both $P_{\mathrm{ET},CO_{2}}$ (estimate = 1.019, *t* = 8.490, *P* < 0.001) and MAP (estimate = 0.107, *t* = 2.243, *P* = 0.026) were significant predictors, with $P_{\mathrm{ET},CO_{2}}$ having a greater influence. The influence of set remained significant (*F* = 2.996, *P *= 0.019), suggesting changes in MCAv over sets.

## 4 DISCUSSION

The purpose of this study was to investigate the effects of the cerebral haemodynamic response to unilateral dynamic upper body RE in healthy individuals. In agreement with our initial hypothesis, the data revealed no significant differences in MCAv_mean_, or any cerebrovascular variables, between the sides contralateral and ipsilateral to the exercising limb. The findings of our study indicate that effects of NVC were not discernible in the MCAv data.

Previous studies report NVC as the primary mechanism increasing contralateral MCAv during static (Braz et al., 2014; Imms et al., 1998) and rhythmic (Giller et al., 2000; Ide et al., 1998; Jorgensen et al., 1993) handgrip exercise. During static RE, NVC is responsible for mediating an increase in CBF during modest increases in MAP (Braz et al., 2014). The increase in MAP during handgrip exercise is gradual and plateaus after ∼90 s (Hirasawa et al., 2016). The slow gradual increase in blood pressure allows for CA to counteract the prevailing blood pressure, thereby facilitating observable NVC‐mediated increases in MCAv contralateral to the exercising limb. However, the present study implemented dynamic upper body RE to assess the contribution of NVC to CBF and observed no difference in contralateral and ipsilateral MCAv_mean_ with respect to the exercising limb. In the present study, we measured blood velocity in a large feed artery (the middle cerebral artery) to estimate the response of blood flow to each hemisphere. The response of the posterior cerebral artery blood velocity to a visual stimulus is a common method of assessing NVC in the occipital lobe, indicating that NVC responses can be detected readily in the large arteries of the circle of Willis. However, in the context of the present experiment, the sinusoidal perturbations in blood pressure generate constantly changing cerebral autoregulatory stimuli, and the adjustments in vessel tone and radius will lag the changes in perfusion pressure (Zhang et al., 1998). It appears from the present data that the changing blood pressure might obfuscate any NVC‐mediated response that is otherwise apparent when MAP is more stable or changing slowly (e.g., during static RE). Furthermore, hypocapnia, as induced by pre‐exercise hyperventilation, reduces within‐exercise MCAv (Romero & Cooke, 2007), indicating the potency of arterial CO_2_ content in regulating CBF (vasoconstriction in this instance) even during rapid changes in perfusion pressure. In the present experiment, the modest within‐RE decrease (2 mmHg) in $P_{\mathrm{ET},CO_{2}}$ is likely to underpin the reduction in MCAv_mean_ across exercise sets. Although a variety of vessels are involved in regulating CBF (Tzeng et al., 2014), because the NVC vascular responses are occurring downstream of the middle cerebral artery in the smaller arteries and arterioles supplying the active brain areas, other methods with greater spatial resolution might be required to identify potential changes in local blood flow between the cerebral hemispheres during dynamic RE.

Although no differences between the contralateral and ipsilateral MCAv_mean_ were apparent, dynamic RE recruiting a small muscle mass generated modest fluctuations in blood pressure that were sufficient to generate bilateral fluctuations in MCAv_mean_. The observed bilateral changes in MCAv_mean_ occurred with much smaller perturbations in blood pressure (and, presumably, cerebral perfusion pressure) than previously reported (Edwards et al., 2002; Perry et al., 2014). Thus, the contraction of a small muscle mass dynamically could still confer beneficial vascular adaptations in both cerebral hemispheres, and unilateral RE could be used therapeutically to increase bilateral shear stress acutely in individuals with hemiplegia or hemiparesis. However, further testing in a clinical setting is required to confirm this.

### 4.1 Limitations

This study used TCD ultrasound to measure MCAv as a proxy for CBF. Although the TCD provides non‐invasive, dynamic and continuous measurement of blood velocity, for MCAv to be an accurate proxy for CBF, middle cerebral artery (MCA) diameter must be unchanged (Ainslie & Hoiland, 2014). Studies examining the effects of hypocapnia on MCA cross‐section area had reported that a reduction in $P_{\mathrm{ET},CO_{2}}$ of ∼12 mmHg decreased MCA cross‐section area by 4% (Coverdale et al., 2014), whereas another observed a non‐significant reduction in MCA cross‐section area of 1.2% when $P_{\mathrm{ET},CO_{2}}$ was reduced by ∼9 mmHg (Verbree et al., 2014). However, the present study produced only small reductions in $P_{\mathrm{ET},CO_{2}}$ (∼2 mmHg). Verbree et al. (2017) found that rhythmic handgrip exercise at 60%–80% MVC produced a 2% decrease in MCA cross‐section area with magnetic resonance imaging, suggestive of exercise‐induced sympathetic vasoconstriction. Therefore, the findings of the present study must be interpreted with caution.

The use of TCD limits the measurement of blood velocity (as a proxy for blood flow) to the large intracranial arteries. Using near‐infrared spectroscopy could further explore localized NVC responses (e.g., in the primary motor cortex). However, concurrent blood volume measurements using near‐infrared spectroscopy would be difficult in the context of the present experiment because the TCD probes are fixed in position using a headband that limits space on the scalp for the placement of additional technologies.

## 5 CONCLUSION

The present findings indicate that during dynamic upper body RE, there were no differences between contralateral and ipsilateral MCAv_mean_. The effects of NVC were not discernible in the MCAv data, and this might be attributable to the overriding influence of $P_{\mathrm{ET},CO_{2}}$ and MAP. Further research is required to investigate how NVC and the regulators of CBF interact during dynamic RE.

## AUTHOR CONTRIBUTIONS

Stephanie Korad, Toby Mündel and Blake Perry contributed to the conceptualization and design of the research. Stephanie Korad and Blake Perry were responsible for data collection. Stephanie Korad, Toby Mündel and Blake Perry were responsible for data analysis, interpretation and drafting of the article. All authors have read and reviewed the article and provided critical feedback. All authors have approved the final version of this manuscript and agree to be accountable for all aspects of the work in ensuring that questions related to the accuracy or integrity of any part of the work are appropriately investigated and resolved. All persons designated as authors qualify for authorship, and all those who qualify for authorship are listed.

## CONFLICT OF INTEREST

None declared.

## Data Availability

The data that support the findings of this study are available from the corresponding author upon reasonable request.
